# Alcohol Advertising Across Spanish and English Television and Radio Networks in New York City

**DOI:** 10.1007/s11524-025-00988-7

**Published:** 2025-07-29

**Authors:** Paola Jiménez Muñoz, Manuel Peña, Alice P. Villatoro, Lu Tang, Melissa J. DuPont-Reyes

**Affiliations:** 1 Department of Psychology, University of Illinois Chicago, 1007 W. Harrison St, 60607 Chicago, IL USA; 2 Public Health Solutions, New York, NY USA; 3 Department of Public Health, Santa Clara University, Santa Clara, CA USA; 4 Department of Communication & Journalism, Texas A&M University, College Station, TX USA; 5 Departments of Sociomedical Sciences and Epidemiology, Columbia University Mailman School of Public Health, New York, NY USA

## Abstract

Latinx residents in New York City experience greater disparities in alcohol use behaviors, chronic liver disease mortality, and other health and legal consequences from high-risk alcohol use compared to non-Latinx White residents. As media-based advertising of alcohol can influence health behaviors, this study aimed to take an “upstream” approach by analyzing rates of alcohol advertising across primetime English- and Spanish-language television networks and radio station broadcasting in New York City during September 7–27, 2022. A systematic content analysis of a randomly drawn, two-week composite sample of primetime YouTube television networks and radio stations revealed significantly higher alcohol advertising rates per hour on Spanish- than English-language media (rate difference across television networks = 4.91, 95% CI = 3.96, 5.85, *p* < 0.05; rate difference across radio stations = 1.86; 95% CI = 1.17, 2.55, *p* < 0.05). Findings underscore disparities in alcohol advertising across diverse media types, disadvantaging consumers of Spanish-language media. Stronger regulation and enforcement of alcohol marketing laws are needed to curb Latinx health inequities.

## Introduction

Health disparities related to substance use across racial and ethnic groups in New York City (NYC) are well-documented. Latinx people, defined here as individuals of Latin American origin or descent, comprise nearly 30% of NYC’s population [1] and disproportionately experience adverse health outcomes, including those linked to high-risk alcohol use [2, 3]. Local public health data reveal that Latinx NYC residents have the highest age-adjusted mortality rates from chronic liver disease and cirrhosis [4], conditions largely driven by excessive alcohol consumption. While non-Latinx White New Yorkers display the highest prevalence of alcohol use disorder (AUD) and binge drinking [5], the medical and legal consequences of high-risk alcohol use behavior for Latinx residents are more severe, including higher alcohol-related hospitalizations and mortality [6]. National data reflect similar patterns [7]. Latina women who engage in high-risk alcohol use face increased odds of intimate partner violence compared to non-Latinx White women [8], while Latino men experience elevated rates of DUI arrests and convictions compared to non-Latinx White men [9]. Moreover, inequitable access to preventative healthcare [2, 3] and financial instability heighten the risk of long-term alcohol dependence and alcohol-related harms among Latinx NYC residents [10].

The US Surgeon General recently emphasized the causal link between alcohol use and cancer risk [11], further underscoring the critical need to address determinants of high-risk alcohol use. Prior studies have shown that “upstream” influences, specifically alcohol advertising in television and radio, can shape consumer behavior to initiate drinking and increase alcohol consumption by promoting perceptions of alcohol as normative and socially desirable—especially among youth and communities with high media exposure [12, 13]. Scrutinizing alcohol advertising practices thus becomes particularly important for Latinx audiences, who have extensive media exposure. In 2023, Latinx households watched an average of 2.16 h of television daily [14], and, in 2021, weekly radio broadcasting reached 86.2% of Latinx adults, indicating its widespread reach among commuters [15]. Latinx populations, especially youth, have been shown to consume more media than non-Latinx Whites and may exhibit limited trust in health promotion messaging [16], potentially reducing their benefit from public health campaigns targeting high-risk alcohol use. Furthermore, alcohol advertisements in traditional media formats (e.g., billboards, magazines, radio, cable news television) are disproportionately broadcast in areas with high Latinx population density [17]. However, alcohol advertising on new digital platforms, such as YouTube TV, and across Spanish- and English-language media remains understudied, leaving disparities in alcohol marketing practices underreported. These gaps could disproportionately impact Latinx media consumers across diverse linguistic, cultural, and technological contexts.

Given the potential role of alcohol marketing in contributing to disproportionate health burdens affecting Latinx populations, this study analyzed alcohol advertising rates across primetime English- and Spanish-language television networks and radio broadcasts in NYC. It offers preliminary evidence of advertisement exposure differences that could inform future regulatory and public health efforts.

## Methods

Data were collected in NYC between September 7 and 27, 2022. Primetime television programming (Monday − Friday 7 − 11 PM EST; Saturday − Sunday 7 − 10 PM EST) from high-viewership Spanish- and English-language networks (Telemundo and Univision; NBC and CBS) [18] was recorded via the largest internet-streaming television service in the US, YouTube TV [19], geolocated to the NYC metro area. A new platform account with no prior activity or profile data was created to minimize personalization and capture advertisement feed intended for a general audience. From this recorded period, a 14-day composite sample of television advertisements was created by randomly selecting each day of the week twice (i.e., two Sundays, two Mondays).

Radio content was collected using similar procedures. Between September 7 and 27, 2022 during typical commuter hours (Monday − Friday 6 − 10 AM and 3 − 7 PM EST), programming from high-listenership Spanish- and English-language radio stations in NYC (La Mega and WBLS) [20] was recorded using stereo equipment. A 10-day composite sample of radio broadcasting was generated by randomly selecting each weekday twice (i.e., two Mondays, two Tuesdays). All recorded radio content was transcribed and uploaded to Dedoose 9.0.17 for analysis.

Methodological details for the television content analysis were previously published [21]. This study focused on alcohol advertising across both media types. Briefly, codebooks were developed for both television and radio content analyses to capture the air date, time, duration, and health-related subject matter of each advertisement, including any promotion of alcoholic beverages. Five trained graduate students (all fluent in English; three in Spanish) coded the samples. Spanish-language content was exclusively coded by fluent speakers. Discrepancies were resolved in weekly meetings with the principal investigator. Interrater reliability was assessed using a random day from each media type, with Cohen’s kappa ranging from 0.75 to 0.99 for television (previously reported) [21] and 0.82 to 0.92 for radio, indicating strong reliability.

Media content coded as alcohol advertisements from the television and radio composite samples constituted the primary unit of analysis. Using STATA/SE 16.1, rate ratios and differences were calculated to compare the rates of alcohol advertisements per hour between Spanish- and English-language media. The rate ratio represents the relative difference in advertisement frequency between the two media languages, while the rate difference quantifies the absolute difference in advertisement rates across Spanish- and English-language programming. Pearson’s chi-square and Fisher’s exact tests assessed differences across media types.

## Results

Table 1 displays frequency, rate ratio, and rate difference estimates in alcohol advertisements in primetime television and radio across Spanish- and English-language networks. The 14-day television sample yielded 9314 total advertisements, with an approximately even split between Spanish- (*n* = 4510) and English-language (*n* = 4804) television networks. Of these, 183 were alcohol advertisements. Spanish-language television aired 157 alcohol advertisements (3.50% of overall Spanish-language television advertisements), compared to 26 alcohol advertisements aired in English-language television networks (0.50% of overall English television advertisements). Differences in alcohol advertising across Spanish- and English-language television were statistically significant: the per-hour rate was 5.72 in Spanish-language television versus 0.82 in English-language television (*p* < 0.05), with a rate difference of 4.91 (95% CI 3.96, 5.85).

**Table 1:** Frequency of alcohol advertising across Spanish- and English-language network primetime television and radio: New York City, September 09, 2022—September 27, 2022

Metric	Primetime television			Primetime radio		
	Full sample	English sample	Spanish sample	Full sample	English sample	Spanish sample
Total ads (*n*%)	9314	4804 (51.58%)	4510 (48.42%)	3468	1992 (57.44%)	1476 (42.56%)
Total alcohol ads (*n*%)	183	26 (0.54%)	157 (3.48%)	28	0 (0.00%)	28 (1.90%)
Alcohol ads rate Per Hour	3.09	0.82	5.72	0.81	0.00	1.86
Alcohol ads rate ratio (95% CI)	n/a	**6.99 (4.62, 10.59)**		n/a	n/a	
Alcohol ads rate difference (95% CI)	n/a	**4.91 (3.96, 5.85)**		n/a	**1.86 (1.17, 2.55)**	

Measures of association in bold are statistically significantly different at *p* < 0.05

*Ad* advertisement, *CI* confidence interval, *n/a* not applicable

The 10-day radio sample yielded 3468 total advertisements, with 1476 advertisements in Spanish- and 1992 advertisements in English-language radio networks. Twenty-eight alcohol advertisements were identified in Spanish-language radio, compared to none in English-language radio. Owing to this stark contrast, the rate difference in frequency of alcohol advertising across Spanish- and English-language radio networks was statistically significant, with 1.86 (95% CI1.17, 2.55) more alcohol advertisements per hour in Spanish-language radio broadcasts than in English-language radio broadcasts (*p* < 0.05).

## Discussion

Spanish-language media in NYC aired significantly more alcohol advertisements compared to English-language media across both primetime YouTube TV and radio. Although alcohol advertisements were not thematically analyzed, a high volume were found embedded within sponsorships of public, family-oriented events in Spanish-language media—despite existing federal guidelines intended to limit underage alcohol advertising exposure through partial content restrictions and audience composition thresholds [22, 23]. This observational pattern, which warrants further investigation, suggests a potential regulatory gap that enables alcohol brands to reach Latinx NYC audiences during primetime hours of heightened familial engagement.

In NYC and the US, alcohol advertising on television and radio is not subject to time-based restrictions or formal content gradation requirements (e.g., by alcohol type or strength) [23]. Further, oversight is primarily managed through voluntary industry self-regulation [23]. This starkly contrasts with more comprehensive regulatory frameworks abroad (e.g., France), which demonstrate the potential of stronger advertising restrictions in reducing alcohol-related health disparities [24].

Alcohol advertising reform therefore remains a critical area of public health inquiry and intervention. Latinx New Yorkers comprised 35% of AUD treatment admissions in 2022 compared to 27.3% in 2018 [25], potentially signaling a rise in AUD rates. Increased recognition of the impact of multilevel contributors to health disparities, such as norms and beliefs reinforced by targeted marketing practices, can help to propel a shift towards regulatory initiatives that curb health inequities among urban Latinx populations.

Some study limitations require discussion. This study is limited by its focus on one city, narrow time-frame, and two media platforms (YouTube TV and radio). Observed advertising may not reflect seasonal or event-driven fluctuations, daily variability, or potential differences between streaming and traditional broadcast delivery. In addition, the study did not collect data on the demographics of media audiences. The evolving landscape of digital marketing warrants investigation into alcohol advertising on social media and additional streaming apps, especially considering the potential for unchecked exposure to minors via algorithm-driven content. Future work should also extend beyond primetime and examine daytime television. Additional inquiry into the themes and persuasive appeals employed in alcohol advertising may enhance understanding of their impact on audience perceptions and behaviors. In sum, future research and policy interventions must continue to spotlight the impact of urban media environments on consumption patterns and related health outcomes among Latinx individuals, with a focus on how corporations and policy may perpetuate, or reduce, these inequities.

## Data Availability

Data that support the findings of this study may be made available at the discretion of the authors upon reasonable request.
